# 3-Amino­benzoic acid–4-nitro­benzoic acid (1/1)

**DOI:** 10.1107/S1600536808037112

**Published:** 2008-11-13

**Authors:** Ching Kheng Quah, Samuel Robinson Jebas, Hoong-Kun Fun

**Affiliations:** a X-ray Crystallography Unit, School of Physics, Universiti Sains Malaysia, 11800 USM, Penang, Malaysia

## Abstract

In the title 1:1 adduct, C_7_H_5_NO_4_·C_7_H_7_NO_2_, the nitro group of the 4-nitro benzoic acid is twisted from the attached ring by 4.40 (8)°. In the crystal, the mol­ecules are linked into ribbon-like structures along [150] and [1
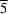
0] *via* O—H⋯O, N—H⋯O, N—H⋯N and C—H⋯O inter­molecular hydrogen bonds.

## Related literature

For the applications of 3-amino­benzoic acid, see; Windholz (1976 ▶). For related structures, see: Bowers *et al.* (2005 ▶); Tonogaki *et al.* (1993 ▶); Voogd *et al.* (1980 ▶).
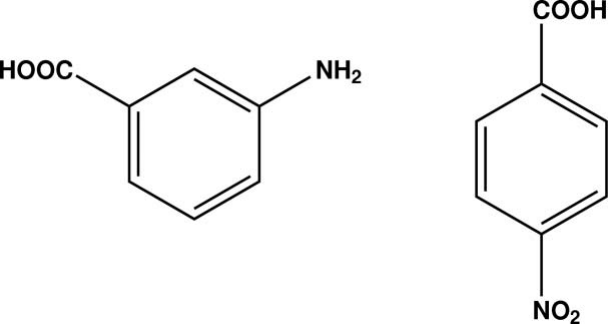


## Experimental

### 

#### Crystal data

C_7_H_5_NO_4_·C_7_H_7_NO_2_
*M* *_r_* = 304.26
Monoclinic, 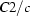

*a* = 25.3707 (8) Å
*b* = 4.9875 (2) Å
*c* = 21.7276 (7) Å
β = 109.230 (2)°
*V* = 2595.93 (16) Å^3^
*Z* = 8
Mo *K*α radiation
μ = 0.12 mm^−1^
*T* = 100.0 (1) K
0.24 × 0.09 × 0.06 mm

#### Data collection

Bruker SMART APEXII CCD area-detector diffractometer
Absorption correction: multi-scan (*SADABS*; Bruker, 2005 ▶) *T*_min_ = 0.971, *T*_max_ = 0.993
15472 measured reflections
3759 independent reflections
2197 reflections with *I* > 2σ(*I*)
*R*_int_ = 0.068

#### Refinement

*R*[*F* ^2^ > 2σ(*F* ^2^)] = 0.068
*wR*(*F* ^2^) = 0.179
*S* = 1.01
3759 reflections
215 parameters
H atoms treated by a mixture of independent and constrained refinement
Δρ_max_ = 0.50 e Å^−3^
Δρ_min_ = −0.36 e Å^−3^

### 

Data collection: *APEX2* (Bruker, 2005 ▶); cell refinement: *SAINT* (Bruker, 2005 ▶); data reduction: *SAINT*; program(s) used to solve structure: *SHELXTL* (Sheldrick, 2008 ▶); program(s) used to refine structure: *SHELXTL*; molecular graphics: *SHELXTL*; software used to prepare material for publication: *SHELXTL* and *PLATON* (Spek, 2003 ▶).

## Supplementary Material

Crystal structure: contains datablocks global, I. DOI: 10.1107/S1600536808037112/ci2714sup1.cif

Structure factors: contains datablocks I. DOI: 10.1107/S1600536808037112/ci2714Isup2.hkl

Additional supplementary materials:  crystallographic information; 3D view; checkCIF report

## Figures and Tables

**Table 1:** Hydrogen-bond geometry (Å, °)

*D*—H⋯*A*	*D*—H	H⋯*A*	*D*⋯*A*	*D*—H⋯*A*
O4—H1*O*4⋯O5^i^	0.89 (4)	1.73 (4)	2.612 (2)	171 (3)
O6—H1*O*6⋯O3^ii^	0.91 (4)	1.75 (4)	2.652 (2)	171 (4)
N2—H1*N*1⋯O2^iii^	1.06 (4)	2.29 (4)	3.309 (3)	161 (3)
N2—H2*N*2⋯O2^iv^	0.90 (3)	2.60 (3)	3.351 (3)	142 (2)
C2—H2*A*⋯O5^v^	0.95	2.58	3.288 (3)	131
C4—H4*A*⋯O6^iv^	0.95	2.55	3.339 (3)	141
C10—H10*A*⋯O1^iii^	0.95	2.57	3.460 (3)	156

Symmetry codes: (i) 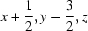; (ii) 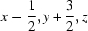; (iii) 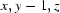; (iv) 
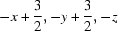
; (v) 
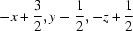
.

**Fig. 1.**
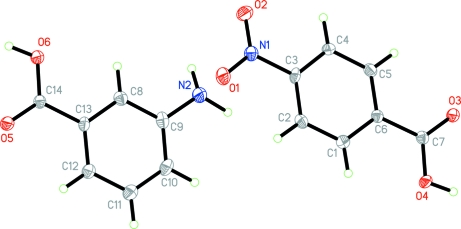
The asymmetric unit of the title compound. Displacement ellipsoids are drawn at the 50% probability level.

**Fig. 2.**
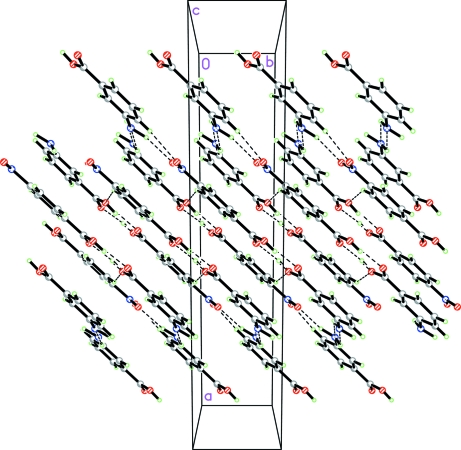
The crystal packing of the title compound, viewed along the c axis. Hydrogen bonds are shown as dashed lines.

## supplementary crystallographic information

### Comment

3-Aminobenzoic acid is used as an intermediate for dyes, pesticides and in other organic synthesis (Windholz, 1976). The crystal structures of 3-aminobenzoic acid (Voogd *et al.*, 1980) and 4-aminobenzoic acid-4-nitrobenzoic acid have been reported (Bowers *et al.*, 2005). As a part of our investigation of the interactions between acids, we report herein the crystal structure of the title compound.

The asymmetric unit (Fig. 1) contains one 3-aminobenzoic acid molecule and one 4-nitrobenzoic acid molecule. The bond lengths and angles of 3-aminobenzoic acid and 4-nitrobenzoic acid are found to have normal values (Voogd *et al.*, 1980; Tonogaki *et al.*, 1993). Both the molecules are almost planar with the maximum deviation from planarity being 0.026 (2) Å for atom O5 of 3-aminobenzoic acid molecule, and 0.078 (2) Å for atom O1 of nitrobenzoic acid molecule. In the 4-nitrobenzoic acid molecule, the nitro group is twisted slightly from the attached ring; the dihedral angle between C1—C6 and O1—O2/C3/N1 planes is 4.40 (8)°.

The crystal packing is consolidated by O—H···O, N—H···O, N—H···N and C—H···O intermolecular hydrogen bonds (Table 1). These hydrogen bonds link the molecules into ribbon like structures along [1 5 0] and [1 5 0] (Fig.2).

### Experimental

3-Aminobenzoic acid and 4-nitrobenzoic acid were mixed in methanol (20 ml) in a 1:1 molar ratio. The clear colourless solution obtained was allowed to evaporate slowly. Colourless crystals were obtained after 2 d.

### Refinement

N and O-bound H atoms were located in a difference Fourier map and were allowed to refine freely. All the other H atoms were placed in calculated positions, with C—H = 0.95 Å, and refined using a riding model, with *U*_iso_(H) = 1.2*U*_eq_(C).

### Crystal data

**Table:**

C_7_H_5_NO_4_·C_7_H_7_NO_2_	*F*_000_ = 1264
*M**_r_* = 304.26	*D*_x_ = 1.557 Mg m^−^^3^
Monoclinic, *C*2/*c*	Mo *K*α radiation λ = 0.71073 Å
Hall symbol: -C 2yc	Cell parameters from 2404 reflections
*a* = 25.3707 (8) Å	θ = 3.0–29.9º
*b* = 4.9875 (2) Å	µ = 0.12 mm^−^^1^
*c* = 21.7276 (7) Å	*T* = 100.0 (1) K
β = 109.230 (2)º	Plate, yellow
*V* = 2595.93 (16) Å^3^	0.24 × 0.09 × 0.06 mm
*Z* = 8	

### Data collection

**Table:**

Bruker SMART APEXII CCD area-detector diffractometer	3759 independent reflections
Radiation source: fine-focus sealed tube	2197 reflections with *I* > 2σ(*I*)
Monochromator: graphite	*R*_int_ = 0.068
*T* = 100.0(1) K	θ_max_ = 30.0º
φ and ω scans	θ_min_ = 2.2º
Absorption correction: multi-scan(SADABS; Bruker, 2005)	*h* = −34→35
*T*_min_ = 0.971, *T*_max_ = 0.993	*k* = −7→7
15472 measured reflections	*l* = −29→30

### Refinement

**Table:**

Refinement on *F*^2^	Secondary atom site location: difference Fourier map
Least-squares matrix: full	Hydrogen site location: inferred from neighbouring sites
*R*[*F*^2^ > 2σ(*F*^2^)] = 0.068	H atoms treated by a mixture of independent and constrained refinement
*wR*(*F*^2^) = 0.179	*w* = 1/[σ^2^(*F*_o_^2^) + (0.087*P*)^2^ + 1.6119*P*] where *P* = (*F*_o_^2^ + 2*F*_c_^2^)/3
*S* = 1.01	(Δ/σ)_max_ = 0.001
3759 reflections	Δρ_max_ = 0.50 e Å^−^^3^
215 parameters	Δρ_min_ = −0.36 e Å^−^^3^
Primary atom site location: structure-invariant direct methods	Extinction correction: none

### Special details

**Table:**

Experimental. The data was collected with the Oxford Cyrosystem Cobra low-temperature attachment.
Geometry. All e.s.d.'s (except the e.s.d. in the dihedral angle between two l.s. planes) are estimated using the full covariance matrix. The cell e.s.d.'s are taken into account individually in the estimation of e.s.d.'s in distances, angles and torsion angles; correlations between e.s.d.'s in cell parameters are only used when they are defined by crystal symmetry. An approximate (isotropic) treatment of cell e.s.d.'s is used for estimating e.s.d.'s involving l.s. planes.
Refinement. Refinement of *F*^2^ against ALL reflections. The weighted *R*-factor *wR* and goodness of fit *S* are based on *F*^2^, conventional *R*-factors *R* are based on *F*, with *F* set to zero for negative *F*^2^. The threshold expression of *F*^2^ > σ(*F*^2^) is used only for calculating *R*-factors(gt) *etc*. and is not relevant to the choice of reflections for refinement. *R*-factors based on *F*^2^ are statistically about twice as large as those based on *F*, and *R*- factors based on ALL data will be even larger.

### Fractional atomic coordinates and isotropic or equivalent isotropic displacement parameters (Å^2^)

**Table:**

	*x*	*y*	*z*	*U*_iso_*/*U*_eq_	
O1	0.82025 (7)	0.6966 (4)	0.14306 (8)	0.0238 (4)	
O2	0.81699 (7)	0.7604 (3)	0.04310 (8)	0.0236 (4)	
O3	1.01559 (7)	−0.2306 (3)	0.07932 (8)	0.0175 (4)	
O4	1.01017 (7)	−0.3174 (3)	0.17847 (8)	0.0183 (4)	
O5	0.57710 (6)	0.7825 (3)	0.18023 (8)	0.0180 (4)	
O6	0.58739 (7)	0.8769 (4)	0.08408 (8)	0.0194 (4)	
N1	0.83479 (8)	0.6430 (4)	0.09588 (9)	0.0178 (4)	
N2	0.73488 (9)	0.2432 (5)	0.06366 (11)	0.0217 (5)	
C1	0.93325 (9)	0.0854 (5)	0.16778 (11)	0.0166 (5)	
H1A	0.9461	−0.0152	0.2070	0.020*	
C2	0.89370 (9)	0.2853 (5)	0.16067 (11)	0.0167 (5)	
H2A	0.8792	0.3241	0.1948	0.020*	
C3	0.87598 (9)	0.4267 (5)	0.10277 (11)	0.0158 (5)	
C4	0.89605 (9)	0.3786 (5)	0.05146 (11)	0.0160 (5)	
H4A	0.8831	0.4800	0.0123	0.019*	
C5	0.93576 (9)	0.1775 (5)	0.05923 (11)	0.0164 (5)	
H5A	0.9503	0.1395	0.0251	0.020*	
C6	0.95413 (8)	0.0322 (5)	0.11703 (10)	0.0136 (5)	
C7	0.99642 (9)	−0.1847 (4)	0.12311 (11)	0.0145 (5)	
C8	0.66801 (9)	0.4869 (5)	0.10050 (11)	0.0172 (5)	
H8A	0.6584	0.5966	0.0627	0.021*	
C9	0.70819 (9)	0.2837 (5)	0.10949 (11)	0.0179 (5)	
C10	0.72180 (10)	0.1297 (5)	0.16603 (12)	0.0199 (5)	
H10A	0.7492	−0.0069	0.1728	0.024*	
C11	0.69646 (9)	0.1707 (5)	0.21230 (12)	0.0203 (5)	
H11A	0.7066	0.0630	0.2505	0.024*	
C12	0.65613 (9)	0.3688 (5)	0.20347 (11)	0.0180 (5)	
H12A	0.6383	0.3962	0.2351	0.022*	
C13	0.64223 (9)	0.5267 (5)	0.14746 (11)	0.0152 (5)	
C14	0.59973 (9)	0.7398 (4)	0.13881 (11)	0.0143 (5)	
H1O4	1.0354 (14)	−0.442 (8)	0.1783 (15)	0.056 (11)*	
H1O6	0.5600 (15)	0.998 (8)	0.0809 (16)	0.063 (11)*	
H1N1	0.7575 (14)	0.064 (8)	0.0643 (16)	0.063 (11)*	
H2N2	0.7158 (12)	0.302 (6)	0.0233 (14)	0.029 (8)*	

### Atomic displacement parameters (Å^2^)

**Table:**

	*U*^11^	*U*^22^	*U*^33^	*U*^12^	*U*^13^	*U*^23^
O1	0.0264 (9)	0.0247 (10)	0.0238 (10)	0.0060 (7)	0.0131 (8)	−0.0027 (7)
O2	0.0266 (9)	0.0214 (10)	0.0227 (9)	0.0058 (7)	0.0079 (7)	0.0042 (7)
O3	0.0198 (8)	0.0161 (9)	0.0190 (8)	0.0049 (7)	0.0095 (7)	0.0019 (7)
O4	0.0226 (9)	0.0164 (9)	0.0182 (9)	0.0086 (7)	0.0098 (7)	0.0039 (7)
O5	0.0213 (8)	0.0155 (9)	0.0190 (9)	0.0037 (7)	0.0090 (7)	0.0012 (7)
O6	0.0222 (9)	0.0206 (9)	0.0185 (9)	0.0069 (7)	0.0108 (7)	0.0035 (7)
N1	0.0180 (9)	0.0171 (10)	0.0187 (10)	0.0008 (8)	0.0067 (8)	−0.0014 (8)
N2	0.0229 (11)	0.0244 (12)	0.0197 (11)	0.0051 (9)	0.0096 (9)	0.0015 (9)
C1	0.0168 (11)	0.0159 (12)	0.0173 (12)	0.0005 (9)	0.0060 (9)	0.0008 (9)
C2	0.0180 (11)	0.0171 (12)	0.0173 (12)	−0.0008 (9)	0.0091 (9)	−0.0019 (9)
C3	0.0123 (10)	0.0125 (12)	0.0227 (12)	0.0023 (8)	0.0057 (9)	−0.0028 (9)
C4	0.0180 (11)	0.0138 (11)	0.0173 (12)	0.0020 (9)	0.0073 (9)	0.0034 (9)
C5	0.0181 (11)	0.0164 (12)	0.0168 (12)	0.0000 (9)	0.0087 (9)	0.0002 (9)
C6	0.0121 (10)	0.0125 (11)	0.0164 (11)	−0.0018 (8)	0.0051 (8)	−0.0007 (9)
C7	0.0139 (10)	0.0132 (12)	0.0169 (12)	−0.0018 (9)	0.0058 (9)	−0.0010 (9)
C8	0.0174 (11)	0.0155 (12)	0.0195 (12)	−0.0017 (9)	0.0073 (9)	−0.0011 (9)
C9	0.0151 (11)	0.0173 (12)	0.0222 (12)	−0.0025 (9)	0.0075 (9)	−0.0058 (9)
C10	0.0172 (11)	0.0151 (12)	0.0251 (13)	0.0029 (9)	0.0040 (10)	−0.0019 (10)
C11	0.0193 (12)	0.0179 (13)	0.0224 (13)	0.0012 (9)	0.0052 (10)	0.0006 (9)
C12	0.0184 (11)	0.0163 (12)	0.0197 (12)	0.0025 (9)	0.0069 (9)	0.0000 (9)
C13	0.0126 (10)	0.0138 (11)	0.0201 (12)	0.0010 (9)	0.0066 (9)	−0.0014 (9)
C14	0.0140 (10)	0.0137 (11)	0.0155 (11)	0.0000 (9)	0.0053 (8)	−0.0014 (9)

### Geometric parameters (Å, °)

**Table:**

O1—N1	1.228 (2)	C3—C4	1.391 (3)
O2—N1	1.233 (2)	C4—C5	1.392 (3)
O3—C7	1.225 (3)	C4—H4A	0.95
O4—C7	1.315 (3)	C5—C6	1.391 (3)
O4—H1O4	0.89 (4)	C5—H5A	0.95
O5—C14	1.235 (3)	C6—C7	1.498 (3)
O6—C14	1.317 (3)	C8—C13	1.396 (3)
O6—H1O6	0.91 (4)	C8—C9	1.405 (3)
N1—C3	1.475 (3)	C8—H8A	0.95
N2—C9	1.391 (3)	C9—C10	1.392 (3)
N2—H1N1	1.06 (4)	C10—C11	1.375 (3)
N2—H2N2	0.90 (3)	C10—H10A	0.95
C1—C2	1.387 (3)	C11—C12	1.390 (3)
C1—C6	1.397 (3)	C11—H11A	0.95
C1—H1A	0.95	C12—C13	1.394 (3)
C2—C3	1.382 (3)	C12—H12A	0.95
C2—H2A	0.95	C13—C14	1.481 (3)
			
C7—O4—H1O4	109 (2)	C1—C6—C7	120.9 (2)
C14—O6—H1O6	111 (2)	O3—C7—O4	124.3 (2)
O1—N1—O2	123.7 (2)	O3—C7—C6	121.5 (2)
O1—N1—C3	118.17 (19)	O4—C7—C6	114.23 (19)
O2—N1—C3	118.17 (18)	C13—C8—C9	119.6 (2)
C9—N2—H1N1	120.1 (18)	C13—C8—H8A	120.2
C9—N2—H2N2	114.7 (18)	C9—C8—H8A	120.2
H1N1—N2—H2N2	114 (3)	N2—C9—C10	120.9 (2)
C2—C1—C6	119.8 (2)	N2—C9—C8	120.5 (2)
C2—C1—H1A	120.1	C10—C9—C8	118.6 (2)
C6—C1—H1A	120.1	C11—C10—C9	121.5 (2)
C3—C2—C1	118.3 (2)	C11—C10—H10A	119.2
C3—C2—H2A	120.8	C9—C10—H10A	119.2
C1—C2—H2A	120.8	C10—C11—C12	120.3 (2)
C2—C3—C4	123.1 (2)	C10—C11—H11A	119.8
C2—C3—N1	118.23 (19)	C12—C11—H11A	119.8
C4—C3—N1	118.6 (2)	C11—C12—C13	119.0 (2)
C3—C4—C5	117.9 (2)	C11—C12—H12A	120.5
C3—C4—H4A	121.0	C13—C12—H12A	120.5
C5—C4—H4A	121.0	C12—C13—C8	120.9 (2)
C6—C5—C4	119.9 (2)	C12—C13—C14	118.7 (2)
C6—C5—H5A	120.0	C8—C13—C14	120.4 (2)
C4—C5—H5A	120.0	O5—C14—O6	122.6 (2)
C5—C6—C1	120.8 (2)	O5—C14—C13	121.7 (2)
C5—C6—C7	118.24 (19)	O6—C14—C13	115.72 (19)
			
C6—C1—C2—C3	0.1 (3)	C5—C6—C7—O4	−178.0 (2)
C1—C2—C3—C4	−0.3 (3)	C1—C6—C7—O4	1.3 (3)
C1—C2—C3—N1	−178.7 (2)	C13—C8—C9—N2	−179.2 (2)
O1—N1—C3—C2	3.6 (3)	C13—C8—C9—C10	−1.0 (3)
O2—N1—C3—C2	−176.6 (2)	N2—C9—C10—C11	178.8 (2)
O1—N1—C3—C4	−175.0 (2)	C8—C9—C10—C11	0.7 (3)
O2—N1—C3—C4	4.9 (3)	C9—C10—C11—C12	0.1 (4)
C2—C3—C4—C5	0.2 (3)	C10—C11—C12—C13	−0.7 (3)
N1—C3—C4—C5	178.7 (2)	C11—C12—C13—C8	0.3 (3)
C3—C4—C5—C6	−0.1 (3)	C11—C12—C13—C14	−179.2 (2)
C4—C5—C6—C1	0.0 (3)	C9—C8—C13—C12	0.5 (3)
C4—C5—C6—C7	179.3 (2)	C9—C8—C13—C14	−179.9 (2)
C2—C1—C6—C5	0.0 (3)	C12—C13—C14—O5	1.0 (3)
C2—C1—C6—C7	−179.3 (2)	C8—C13—C14—O5	−178.6 (2)
C5—C6—C7—O3	1.7 (3)	C12—C13—C14—O6	−178.2 (2)
C1—C6—C7—O3	−179.0 (2)	C8—C13—C14—O6	2.2 (3)

### Hydrogen-bond geometry (Å, °)

**Table:**

*D*—H···*A*	*D*—H	H···*A*	*D*···*A*	*D*—H···*A*
O4—H1O4···O5^i^	0.89 (4)	1.73 (4)	2.612 (2)	171 (3)
O6—H1O6···O3^ii^	0.91 (4)	1.75 (4)	2.652 (2)	171 (4)
N2—H1N1···O2^iii^	1.06 (4)	2.29 (4)	3.309 (3)	161 (3)
N2—H2N2···O2^iv^	0.90 (3)	2.60 (3)	3.351 (3)	142 (2)
C2—H2A···O5^v^	0.95	2.58	3.288 (3)	131
C4—H4A···O6^iv^	0.95	2.55	3.339 (3)	141
C10—H10A···O1^iii^	0.95	2.57	3.460 (3)	156

Symmetry codes: (i) *x*+1/2, *y*−3/2, *z*; (ii) *x*−1/2, *y*+3/2, *z*; (iii) *x*, *y*−1, *z*; (iv) −*x*+3/2, −*y*+3/2, −*z*; (v) −*x*+3/2, *y*−1/2, −*z*+1/2.
